# Body mass index may predict the response to ipilimumab in metastatic melanoma: An observational multi-centre study

**DOI:** 10.1371/journal.pone.0204729

**Published:** 2018-10-01

**Authors:** Georg Richtig, Christoph Hoeller, Martin Wolf, Ingrid Wolf, Barbara M. Rainer, Günter Schulter, Markus Richtig, Martin R. Grübler, Anna Gappmayer, Thomas Haidn, Julian Kofler, Rainer Huegel, Bernhard Lange-Asschenfeldt, Martin Pichler, Stefan Pilz, Akos Heinemann, Erika Richtig

**Affiliations:** 1 Otto Loewi Research Center, Pharmacology Section, Medical University of Graz, Graz, Austria; 2 Department of Dermatology, Medical University of Graz, Graz, Austria; 3 Department of Dermatology, Medical University of Vienna, Vienna, Austria; 4 Department of Dermatology, State Hospital Wiener Neustadt, Wiener Neustadt, Austria; 5 Department of Psychology, Biological Psychology Unit, University of Graz, Graz, Austria; 6 Swiss Cardiovascular Center Bern, Department of Cardiology, Bern University Hospital, Bern, Switzerland; 7 Department of Internal Medicine, Division of Endocrinology and Diabetology, Medical University of Graz, Graz, Austria; 8 Department of Dermatology and Venereology, State Hospital Klagenfurt, Klagenfurt am Wörthersee, Austria; 9 Skin Cancer Center Charité, Department of Dermatology and Allergy, Charité-Universitätsmedizin Berlin, Berlin, Germany; 10 Division of Oncology, Medical University Graz, Graz, Austria; 11 Department of Experimental Therapeutics, The University of Texas MD Anderson Cancer Center, Houston, Texas, United States of America; University of Queensland Diamantina Institute, AUSTRALIA

## Abstract

**Introduction:**

Immunotherapy is a well-established treatment option in patients with metastatic melanoma. However, biomarkers that can be used to predict a response in these patients have not yet been found, putting patients at risk of severe side effects.

**Methods:**

In this retrospective analysis, we investigated the association between the body mass index and ipilimumab treatment response in patients with metastatic melanoma. Patients with metastatic melanoma who received a monotherapy of up to 4 doses of ipilimumab (3 mg/kg) every 3 weeks from 2011 to 2014 in three major hospitals in Austria were included. Patients were classified into two groups: normal group (BMI<25) and overweight group (BMI≥25).

**Results:**

40 patients had a normal BMI, and 36 had a BMI above normal. Patients with a BMI that was above normal showed significantly higher response rates (*p* = 0.024, χ^2^), and lower likelihood of brain metastases (*p* = 0.012, χ^2^). No differences were found between both groups with respect to gender (*p* = 0.324, χ^2^), T-stage (p = 0.197, χ^2^), or the occurrence of side effects (*p* = 0.646, χ^2^). Patients with a BMI above normal showed a trend towards longer overall survival (*p* = 0.056, Log-Rank), but no difference was found regarding progression-free survival (*p* = 0.924, Log-Rank).

**Conclusions:**

The BMI correlated with the response to ipilimumab treatment in a cohort of metastatic melanoma patients.

## Introduction

The introduction of the immune checkpoint inhibitors ipilimumab (monoclonal antibody [mAb] against CTLA-4) and nivolumab/pembrolizumab (mAb against PD-1), as well as the combination of ipilimumab and nivolumab, represented a breakthrough in the treatment of metastatic melanoma and many other cancer types.[1,2] Because these drugs lead to long-term responses in many patients, they have been approved for the treatment of metastatic disease in melanoma. Ipilimumab and nivolumab have additionally been approved for the adjuvant treatment of high-risk melanoma in the U.S. and, recently, nivolumab, for adjuvant treatment in Europe.[3,4] However, despite promising data on the efficacy of immune checkpoint inhibitors, several knowledge gaps and challenges regarding the optimal application of these therapeutic agents still exist at present.[5]

Obesity is characterized by a self-sustaining inflammatory response, referred to as ‘meta-inflammation’.[6] It is also accompanied by increased amounts of infiltration of CD4^+^ and CD8^+^ T-cells, B-cells, and eosinophils into white adipose tissues.[7] Moreover, lipogenesis in primary lymphoid organs leads to a disturbance of the immune surveillance, which in turn might favour cancer and autoimmunity.[7] Obesity is a world-wide problem, and one cause of obesity in the developed world is the low-fibre diet, which leads to increased Body Mass Index (BMI) and alterations in the microbial diversity and composition.[8] Recent studies have demonstrated that changes in microbial diversity have an impact on the PD-L1/2 expression of immune cells and might influence the efficacy of CTLA-4 and PD-1 immunotherapy.[9–12]

We considered this potential correlation between obesity and its changes in microbial diversity and inflammation together with the increasing application of immune checkpoint inhibitors in the treatment of melanoma patients and hypothesized that a higher BMI could be associated with a poor response to ipilimumab treatment in patients with metastatic melanoma. Therefore, we tested this hypothesis in a retrospective multi-centre analysis to determine whether BMI is associated with the treatment response, progression free survival (PFS), and overall survival (OS) rates of melanoma patients treated with ipilimumab.

## Material and methods

We performed a retrospective study in patients who received treatment with ipilimumab monotherapy for metastatic melanoma from 2011 to 2014 in three hospitals in Austria. Patients were treated at the Department of Dermatology at the Medical University of Graz, the Department of Dermatology of the State Hospital in Klagenfurt, and the Department of Dermatology of the State Hospital in Wiener Neustadt. Patients of all ages, regardless of their mutational status (*BRAF*, *NRAS*, *KIT*), were eligible for study inclusion, if they had a detailed medical history including data on the primary tumour, mutational status, history of metastases and outcome, including a full set of follow-up data. Treatment surveillance was performed at the respective study sites. Up to 4 doses of ipilimumab were administered intravenously at the approved dose of 3 mg/kg per dose every 3 weeks. Clinical assessment, including laboratory examinations and computed tomography (CT) scans, was conducted, and treatment outcomes were documented. OS was calculated from the date of treatment induction to the date of death by any cause. PFS was calculated from the date of treatment initiation to the date of progression as documented by imaging (CT-scan), clinical examination, or death. An objective response was assessed according to response evaluation criteria in solid tumours (RECIST 1.1) or death.[13] Those alive and without progression were censored at the last follow-up. The first response was assessed three months (mean: 88 days; SD ± 29 days) after the first ipilimumab infusion and every three months thereafter and classified as complete response (CR), partial response (PR), stable disease (SD), or progressive disease (PD). For the purpose of this study, CR, PR, and SD were considered to be clinical benefits. In cases of pseudo-progression according to the Immune-related Response Criteria (irRC)[14], an additional staging was performed six–eight weeks afterwards to either confirm or refute the results. Side effects were reported as adverse events (Grade 1–4 according to the CTCAE criteria version 4.0). The BMI was calculated as weight in kilograms divided by the square of the height in meters (kg/m^2^) on day 1 immediately before the first infusion of ipilimumab therapy. To classify the patients in our analysis, we used a modified BMI classification, which was based on the BMI classification proposed by the World Health Organization.[15] The patients were dichotomized into two groups, i.e., in a patient group with a BMI lower than 25 kg/m^2^ (normal weight group) and patient group with a BMI higher than or exactly 25 kg/m^2^ (overweight group).

The 2009 edition of the American Joint Committee on Cancer (AJCC) TNM system was used to assess the stage of disease classification.[16]

The retrospective analysis was approved by the local ethics committee of the Medical University of Graz (ID: 29–202 ex 16/17).

### Statistical analysis of clinicopathological parameters

The normal distribution of continuous variables was assessed by visually inspecting the histograms, Q-Q plots, and the skewness and kurtosis (-1 to +1). Variables displaying a non-normal (skewed-) distribution were log10-transformed before their inclusion in parametric statistical tests. Fisher’s exact test, χ2 test, and the Mann-Whitney *U* tests were used where appropriate to analyse BMI in relation to each clinicopathological parameter. Baseline characteristics were reported as means and their standard deviation, as medians with interquartile range, or as percentages. The primary endpoint of the study was the objective response to treatment in patients with a BMI above and below 25 kg/m^2^. Secondary outcomes were the overall survival and progression free survival rates. Event-time distributions and the 5-year overall survival of patients were calculated using the Kaplan-Meier method followed by the log-rank test. A two-sided alpha level of 0.05 was considered significant. All statistical analyses were performed using SPSS version 23.0 software (SPSS, Chicago, IL).

## Results

Seventy-six patients were included in this retrospective analysis: 30 female patients (39.5%), 13 (43.4%) of whom were overweight, and 46 (60.5%) male patients, 23 (50.0%) of whom were overweight (full baseline characteristics are shown in S1 Table).

In the entire cohort, the mean (SD; min to max in kg/m^2^) BMI was 25.6 (± 5.6; 18.0 to 59.1), with a BMI of 29.4 (± 5.9; 25.1 to 59.2) in the overweight group, and of 22.2 (± 1.8; 18.0 to 24.7) for the normal BMI group, respectively. Baseline characteristics stratified by BMI groups are shown in Table 1. Lymph node and spleen metastases were added as parts of the peripheral lymphoid organ system.

**Table 1:** Patients’ characteristics divided by Body-Mass-Index.

	Normal (*BMI < 25*) (*n* = 40)	Overweight (*BMI ≥ 25*) (*n* = 36)	p
*Gender*			0.324^a^
Male	23 (57,5%)	23 (63.9%)	
Female	17 (42,5%)	13 (36.1%)	
*M1c*			**0.048**^a^
no	10 (25.0%)	16 (47.1%)	
yes	30 (75.0%)	18 (52.9%)	
*Adjuvant Therapy*			0.467^a^
Yes	20 (50.0%)	21 (58.3%)	
No	20 (50.0%)	15 (41.7%)	
*BRAF*			0.366^a^
Wild-type	19 (47.5%)	20 (55.6%)	
Mutated	18 (45.0%)	11 (30.6%)	
Not assessed	3 (7.5%)	17 (47.2%)	
*Breslow thickness* *(mm*, *SD)*	4.08 ± 3.92	7.72 ± 9.30	0.447^b^
*Side effects*			0.646^a^
*yes*	19 (47.5%)	19 (52.8%)	
*no*	21 (52.5%)	17 (47.2%)	
*Brain metastases*			**0.012**^a^
yes	13 (32.5%)	3 (8.6%)	
no	27 (67.5%)	32 (91.4%)	
*Spleen metastases*			0.677^c^
yes	4 (10.0%)	2 (5.6%)	
no	36 (90.0%)	34 (94.4%)	
*Lymph node metastases*			0.978
yes	29 (72.5%)	26 (72.2%)	
no	11 (27.5%)	10 (27.8%)	
*LDH at therapy start*			**0.029**^a^
normal	19 (47.5%)	24 (72.7%)	
elevated	21 (52.5%)	9 (27.3%)	
*S100 at therapy start*			**0.043**^a^
normal	16 (41.0%)	22 (64.7%)	
elevated	23 (59.0%)	12 (35.3%)	
*CRP at therapy start*			0.154^a^
normal	16 (40.0%)	8 (24.2%)	
elevated	24 (60.0%)	25 (75.8%)	
*ECOG performance status*			0.256^a^
0	17 (42.5%)	20 (55.6%)	
≥ 1	23 (57.5%)	16 (44.4%)	

^a^ = χ^2^ test;

^b^ = Mann-Whitney *U* test;

^c^ = Fisher’s exact test

No difference was found between both groups regarding gender (*p* = 0.324, χ^2^ test), T stage (*p* = 0.197, χ^2^ test), *BRAF* mutation status (*p* = 0.366, χ^2^ test), ECOG performance status (*p* = 0.256, χ^2^ test), tumour thickness (*p* = 0.447, Mann-Whitney *U* Test), immune-related side effects (*p* = 0.498, χ^2^ test), or metastases in spleen (*p* = 0.677, Fisher’s exact test), lymph nodes (*p* = 0.978, χ^2^ test), NLR (*p* = 0.318, Fisher’s exact test), or elevation of eosinophils under therapy (*p* = 0.165, χ^2^ test). However, compared to the normal weight group, patients with a BMI over 25 kg/m^2^ were significantly less likely to have brain metastases (***p* = 0.012**, χ^2^ test), less likely to have M1c stage (***p* = 0.048**, χ^2^ test), had a significantly higher prevalence of normal LDH levels (***p* = 0.029**, χ^2^ test), and normal S100 levels at the start of therapy (***p* = 0.043**, χ^2^ test). Normal LDH levels at the therapy start were not associated with a higher likelihood of treatment response (*p* = 0.156, χ^2^ test), and no correlation between LDH levels and treatment response was found (*p* = 0.186, Mann-Whitney *U*).

### Longitudinal analysis

**Fig 1:**
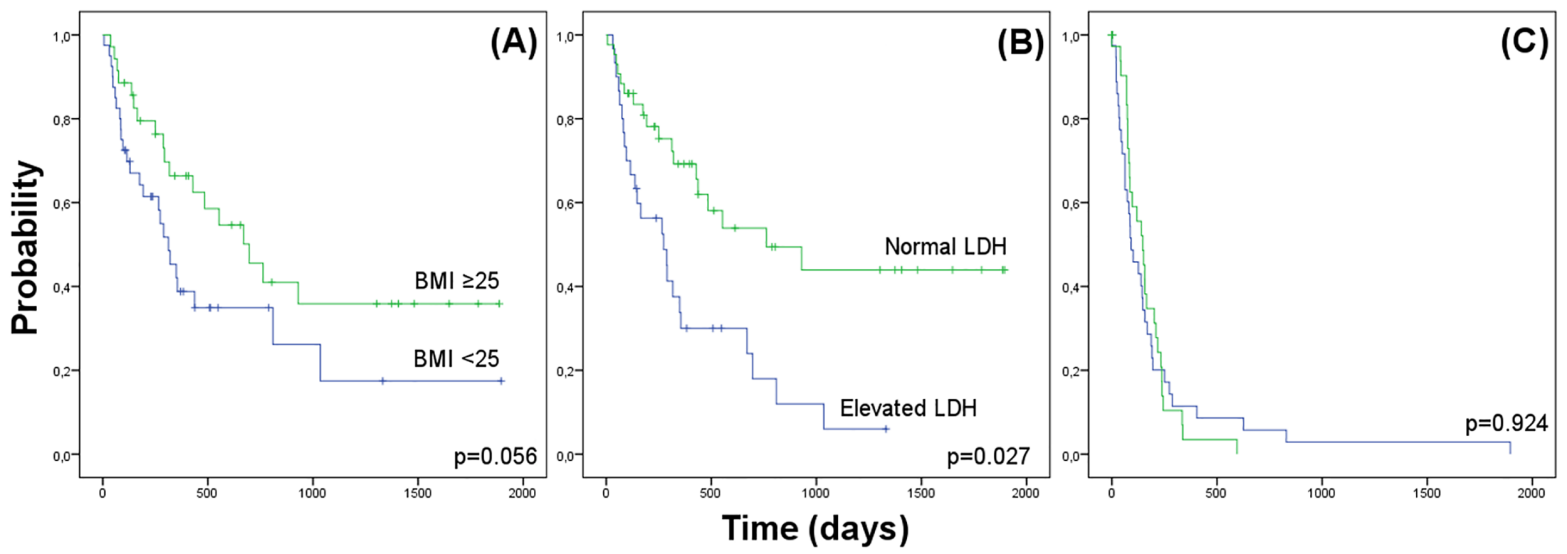
Kaplan-Meier-Plots of patients treated with ipilimumab for metastatic melanoma according to different parameters. Overall survival of patients treated with ipilimumab grouped by (A) BMI and (B) LDH levels. Progression-free survival rates of patients treated with ipilimumab, grouped by BMI (C).

In the longitudinal analyses, the overweight group had a significantly higher likelihood of showing an objective response to ipilimumab treatment (***p* = 0.024**, χ^2^ test) compared to the normal weight group. Overweight patients showed a non-significant trend towards longer overall survival (*p* = 0.056, log-rank test; hazard ratio [HR] = 1.81, Cl_95%_ = 0.98–3.33), and no difference was found with regard to PFS (*p* = 0.924, log-rank test; HR = 1.03, CI_95%_ = 0.62–1.70). Normal LDH levels at therapy initiation were associated with significantly longer OS (***p* = 0.027**, log-rank test) but not with PFS (*p* = 0.217, log-rank test) (shown in detail in Fig 1). Decreases in the numbers of eosinophils (***p* = 0.027**, log-rank test) (S1A Fig) and NLR > 5 (***p* = 0.029**, log-rank test) (S1B Fig) during therapy were statistically significantly associated with shorter OS but not with PFS (*p* = 0.791; log-rank test, and *p* = 0.538; log-rank test), respectively.

### Multivariate analysis

Binary logistic regression analysis was performed between the overweight group (BMI ≥25) and the treatment response group, controlling for the common prognostic markers age, sex, visceral metastases (M1c), ECOG, and LDH levels at the start of immunotherapy. After controlling for the effects of all other prognostic markers, the association between the independent variable ‘overweight’ and the dependent variable ‘treatment response’ was nearly significant at the 0.05 level (*p* = 0.084; Odds ratio = 2.75; Cl_95%_ = 0.87–8.66; see Table 2). To further estimate the effect of obesity on the response rate by simultaneously controlling for the different stages of disease (M1a, M1b, M1c), a logistic regression was calculated. The results clearly showed that obesity had a significant effect on the response rate irrespective of the respective stage of disease (*p* = 0.037; OR = 2.80, CI_95%_ = 1.06–7.39). On the contrary, the stage of disease in itself was not significantly related to the response type (*p* = 0.963).

**Table 2:** Results of the binary logistic regression for parameters included in the analysis.

Parameter	Odds ratio	*p*-value	95% CI	
			Lower	Upper
Age	0.983	0.496	0.936	1.032
Sex	0.405	0.127	0.127	1.294
M1c	0.875	0.831	0.258	2.972
LDH	0.999	0.529	0.996	1.002
ECOG	5.240	**0.005**	1.651	16.630
Overweight	2.751	0.084	0.874	8.658

## Discussion

Herein, we report on an observation of a positive correlation between the BMI and response to CTLA-4 mAb ipilimumab in a cohort of patients from three Austrian centres.

In the literature, higher BMI has been associated with a higher likelihood of developing melanoma but also with a better outcome for metastatic diseases in different cancer forms.[17,18] In our cohort, overweight patients showed a significantly better treatment response compared to patients with normal weight, but only a trend towards longer overall survival. No difference was observed regarding the PFS between the groups. Although the study by McQuade et al. showed improved PFS for obese patients in their immunotherapy cohort, their study included patients treated with both ipilimumab and dacarbazine, whereas our patients were treated with ipilimumab as monotherapy.[19] Therefore, the beneficial effect of additional chemotherapy with ipilimumab on PFS cannot be ruled out. Recently, Daly et al. also speculated that ipilimumab might augment existing systemic inflammation in patients, because they observed an increased incidence of high-grade AEs in patients with sarcopenia and low muscle attenuation.[20] Since BMI in our cohort was only assessed at the start of therapy, changes in body composition by patients might have had an influence on PFS. Unlike McQuade et al., we did not observe a significant improvement in OS, which might be due to the underpowered study design.

LDH levels are one of the most important predicting markers for outcome in immune therapy. In our study, obesity was associated with normal LDH levels at the start of therapy. This might be interpreted as a more effective prognostic group, but in a meta-inflammatory state, where the immune system is highly active—and at some point exhausted—as occurs in obesity, dying cancer cells might release lower levels of LDH. Therefore, it might be possible that, on the one hand, a potential lower tumour burden (lower LDH levels at therapy start) might have been responsible for the improved treatment response in our overweight patients, whereas, on the other hand, meta-inflammatory conditions in the host at the beginning of therapy could have led to the same results. Because we found no difference in the PFS between both groups, we could speculate that the treatment effect might have been lost due to immune exhaustion and changes in the tumour microenvironment (early secondary resistance).[21]

All in all, these results might be viewed as puzzling, but the meta-inflammation due to obesity might only play a role in the metastatic tumour environment and in the local response to treatment. The ECOG performance status and CRP did not differ between the groups, indicating that patients were not pre-selected at the start of therapy. Regarding the comparability of our study cohort with other published cohorts, we demonstrated that an increase in the eosinophil count was associated with better overall survival as had been shown by Delyon et al.[22] In addition, a positive neutrophil-to-lymphocyte ratio was associated with better overall survival, a finding that has been reported by several other studies.[23–25]

The strength of this study was that it was a multi-centre study carried out in three major hospitals in Austria with patients treated under ‘real world’ conditions as part of routine, daily clinical practice. The major limitation of this study, however, is its retrospective character and limited number of patients. These limitations did not allow us to determine whether the BMI can be used as a predictive or a prognostic marker or not. However, as our results suggest that it can be used to identify patients who would be more likely to benefit from immunotherapy in advance, we strongly encourage authors of prospective studies to include BMI as a parameter in their analyses, particularly since it can easily and promptly be calculated.

In conclusion, the BMI can potentially be used to predict the response of metastatic melanoma patients to ipilimumab treatment. Correlations between obesity, the microbial flora, and immunotherapy in cancer patients is of major interest, as an increase in weight and, thus, in BMI leads changes in microbial diversity and composition. The question of whether nutritional changes have an impact on responses to immunotherapy is especially tantalizing.

## Supporting information

S1 Table Clinico-pathological characteristics of patients in this study. (DOCX)

S1 Fig Kaplan-Meier-Plots of patients treated with ipilimumab for metastatic melanoma divided by blood eosinophils count (A) and blood Neutrophil-To-Lymphocyte ratio (B). (PNG)

## References

[1] O’Day SJ, Hamid O, Urba WJ. Targeting cytotoxic T-lymphocyte antigen-4 (CTLA-4): a novel strategy for the treatment of melanoma and other malignancies. Cancer. 2007;110: 2614–27. 10.1002/cncr.23086 18000991

[2] Wolchok JD, Kluger H, Callahan MK, Postow MA, Rizvi NA, Lesokhin AM, et al Nivolumab plus ipilimumab in advanced melanoma. N Engl J Med. 2013;369: 122–33. 10.1056/NEJMoa1302369 23724867 PMC5698004

[3] Eggermont AMM, Chiarion-Sileni V, Grob J-J, Dummer R, Wolchok JD, Schmidt H, et al Prolonged Survival in Stage III Melanoma with Ipilimumab Adjuvant Therapy. N Engl J Med. 2016;375: 1845–1855. 10.1056/NEJMoa1611299 27717298 PMC5648545

[4] Weber J, Mandala M, Del Vecchio M, Gogas HJ, Arance AM, Cowey CL, et al Adjuvant Nivolumab versus Ipilimumab in Resected Stage III or IV Melanoma. N Engl J Med. 2017; 10.1056/NEJMoa1709030 28891423

[5] Richtig G, Pichler M. Prediction of Response in Melanoma Therapy by Systemic Inflammatory Response—One Size Fits Not All. EBioMedicine. 2017;18: 13–14. 10.1016/j.ebiom.2017.03.032 28366295 PMC5405174

[6] Mirsoian A, Murphy WJ. Obesity and cancer immunotherapy toxicity. Immunotherapy. 2015;7: 319–322. 10.2217/imt.15.12 25917623 PMC4787259

[7] Kanneganti T-D, Dixit VD. Immunological complications of obesity. Nat Immunol. 2012;13: 707–712. 10.1038/ni.2343 22814340

[8] Lynch S V., Pedersen O. The Human Intestinal Microbiome in Health and Disease. Phimister EG, editor. N Engl J Med. 2016;375: 2369–2379. 10.1056/NEJMra1600266 27974040

[9] Trompette A, Gollwitzer ES, Yadava K, Sichelstiel AK, Sprenger N, Ngom-Bru C, et al Gut microbiota metabolism of dietary fiber influences allergic airway disease and hematopoiesis. Nat Med. 2014;20: 159–166. 10.1038/nm.3444 24390308

[10] Vétizou M, Pitt JM, Daillère R, Lepage P, Waldschmitt N, Flament C, et al Anticancer immunotherapy by CTLA-4 blockade relies on the gut microbiota. Science. 2015;350: 1079–84. 10.1126/science.aad1329 26541610 PMC4721659

[11] Sivan A, Corrales L, Hubert N, Williams JB, Aquino-Michaels K, Earley ZM, et al Commensal Bifidobacterium promotes antitumor immunity and facilitates anti-PD-L1 efficacy. Science (80-). 2015;350: 1084–1089. 10.1126/science.aac4255 26541606 PMC4873287

[12] Snyder A, Pamer E, Wolchok J. IMMUNOTHERAPY. Could microbial therapy boost cancer immunotherapy? Science. 2015;350: 1031–2. 10.1126/science.aad7706 26612936

[13] Eisenhauer EA, Therasse P, Bogaerts J, Schwartz LH, Sargent D, Ford R, et al New response evaluation criteria in solid tumours: Revised RECIST guideline (version 1.1). Eur J Cancer. 2009;45: 228–247. 10.1016/j.ejca.2008.10.026 19097774

[14] Wolchok JD, Hoos A, O’Day S, Weber JS, Hamid O, Lebbe C, et al Guidelines for the Evaluation of Immune Therapy Activity in Solid Tumors: Immune-Related Response Criteria. Clin Cancer Res. 2009;15: 7412–7420. 10.1158/1078-0432.CCR-09-1624 19934295

[15] Ligibel JA, Alfano CM, Courneya KS, Demark-Wahnefried W, Burger RA, Chlebowski RT, et al American Society of Clinical Oncology Position Statement on Obesity and Cancer. J Clin Oncol. 2014;32: 3568–3574. 10.1200/JCO.2014.58.4680 25273035 PMC4979237

[16] Balch CM, Gershenwald JE, Soong S-J, Thompson JF, Atkins MB, Byrd DR, et al Final version of 2009 AJCC melanoma staging and classification. J Clin Oncol. 2009;27: 6199–206. 10.1200/JCO.2009.23.4799 19917835 PMC2793035

[17] Tsang NM, Pai PC, Chuang CC, Chuang WC, Tseng CK, Chang KP, et al Overweight and obesity predict better overall survival rates in cancer patients with distant metastases. Cancer Med. 2016;5: 665–75. 10.1002/cam4.634 26811258 PMC4831285

[18] Skowron F, Bérard F, Balme B, Maucort-Boulch D. Role of obesity on the thickness of primary cutaneous melanoma. J Eur Acad Dermatology Venereol. 2015;29: 262–269. 10.1111/jdv.12515 24750303

[19] McQuade JL, Daniel CR, Hess KR, Mak C, Wang DY, Rai RR, et al Association of body-mass index and outcomes in patients with metastatic melanoma treated with targeted therapy, immunotherapy, or chemotherapy: a retrospective, multicohort analysis. Lancet Oncol. 2018;19: 310–322. 10.1016/S1470-2045(18)30078-0 29449192 PMC5840029

[20] Daly LE, Power DG, O’Reilly Á, Donnellan P, Cushen SJ, O’Sullivan K, et al The impact of body composition parameters on ipilimumab toxicity and survival in patients with metastatic melanoma. Br J Cancer. 2017;116: 310–317. 10.1038/bjc.2016.431 28072766 PMC5294486

[21] Shirakawa K, Yan X, Shinmura K, Endo J, Kataoka M, Katsumata Y, et al Obesity accelerates T cell senescence in murine visceral adipose tissue. J Clin Invest. 2016;126: 4626–4639. 10.1172/JCI88606 27820698 PMC5127667

[22] Delyon J, Mateus C, Lefeuvre D, Lanoy E, Zitvogel L, Chaput N, et al Experience in daily practice with ipilimumab for the treatment of patients with metastatic melanoma: an early increase in lymphocyte and eosinophil counts is associated with improved survival. Ann Oncol Off J Eur Soc Med Oncol. 2013;24: 1697–703. 10.1093/annonc/mdt027 23439861

[23] Cassidy MR, Wolchok RE, Zheng J, Panageas KS, Wolchok JD, Coit D, et al Neutrophil to Lymphocyte Ratio is Associated With Outcome During Ipilimumab Treatment. EBioMedicine. 2017;18: 56–61. 10.1016/j.ebiom.2017.03.029 28356222 PMC5405176

[24] Ferrucci PF, Gandini S, Battaglia A, Alfieri S, Di Giacomo AM, Giannarelli D, et al Baseline neutrophil-to-lymphocyte ratio is associated with outcome of ipilimumab-treated metastatic melanoma patients. Br J Cancer. 2015;112: 1904–1910. 10.1038/bjc.2015.180 26010413 PMC4580390

[25] Ferrucci PF, Ascierto PA, Pigozzo J, Del Vecchio M, Maio M, Antonini Cappellini GC, et al Baseline neutrophils and derived neutrophil-to-lymphocyte ratio: prognostic relevance in metastatic melanoma patients receiving ipilimumab. Ann Oncol. 2016;27: 732–738. 10.1093/annonc/mdw016 26802161

