# Supplementary material for: Body mass index may predict the response to ipilimumab in metastatic melanoma: An observational multi-centre study
Source: PLoS One. 2018 Oct 1;13(10):e0204729. doi: 10.1371/journal.pone.0204729 (PMC6166940; doi:10.1371/journal.pone.0204729)
Supplement: S1 Table — (DOCX) [file pone.0204729.s001.docx]

**S1 Table.**

| Clinicopathological parameter | No. of patients (n=76)(%) |
| --- | --- |
| **Sex** | |
| Male Female | 46/76 (60.5%) 30/76 (39.5%) |
| **Body-Mass-Index** | |
| Normal BMI (< 25) Overweight (≥ 25) | 40/76 (52.6%) 36/76 (47.4%) |
| **Tumor stage** | |
| T1 T2 T3 T4 Tx | 8/76 (10.5%) 14/76 (18.4%) 17/76 (22.4%) 23/76 (30.3%) 14/76 (18.4%) |
| N0 N1 N2 N3 | 17/76 (22.4%) 10/76 (13.2%) 8/76 (10.5%) 38/76 (50.0%) |
| **M1c** | |
| Yes No | 48/76 (63.2%) 24/76 (31.6%) |
| **Adjuvant therapy** | |
| no yes | 35/76 (46.1%) 41/76 (53.9%) |
| **Mutation status** | |
| *BRAF^wt^ BRAF^mt^* not assessed | 39/76 (51.3%) 29/76 (38.2%) 8/76 (10.5%) |
| **ECOG Performance status** | |
| ECOG 0 ECOG ≥ 1 | 37/76 (48.7%) 39/76 (51.3%) |
| **LDH** | |
| < 240 U/l (normal) ≥ 240 U/l (elevated) | 43/76 (56.6%) 30/76 (39.5%) |
| **CRP** | |
| < 5 mg/l (normal) ≥ 5 mg/l (elevated) | 49/76 (64.5%) 24/76 (31.6%) |
| **S100** | |
| < 0.100 µg/l (normal) ≥ 0.100 µg/l (elevated) | 38/76 (50.0%) 35/76 (46.1%) |

Missing values are not shown explicitly but are the difference to the given total number and to hundred percent.
