# Supplementary figures and images for: Body mass index may predict the response to ipilimumab in metastatic melanoma: An observational multi-centre study
Source: PLoS One. 2018 Oct 1;13(10):e0204729. doi: 10.1371/journal.pone.0204729 (PMC6166940; doi:10.1371/journal.pone.0204729)

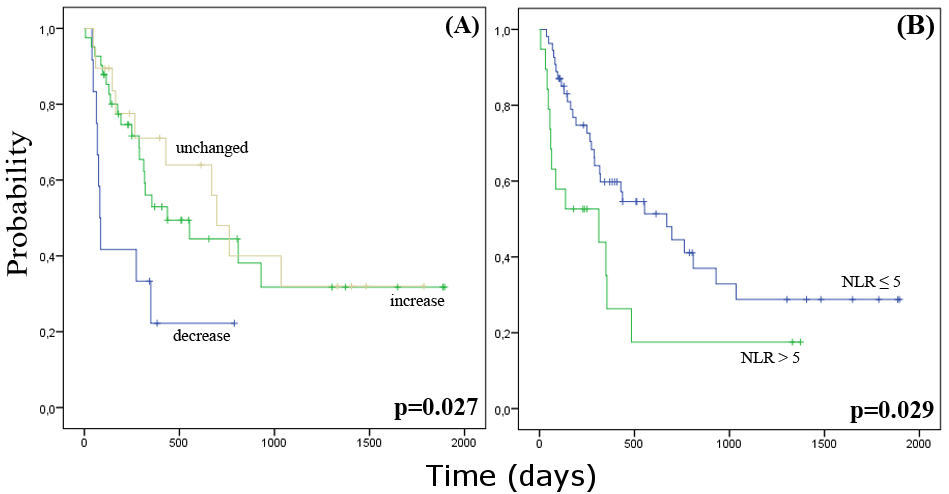

Supplement: S1 Fig — (PNG) [file pone.0204729.s002.png]
